# Association between vitamin D levels and mortality in hemodialysis patients: a cohort study

**DOI:** 10.1080/0886022X.2020.1735415

**Published:** 2020-03-03

**Authors:** Maryanne Machado da Silva Canhos, Rogério Carvalho de Oliveira, Luis Gustavo Modelli de Andrade, Jacqueline Costa Teixeira Caramori, Pasqual Barretti, Luis Cuadrado Martin

**Affiliations:** Division of Nephrology, Department of Internal Medicine, Botucatu Medical School, São Paulo State University (UNESP), Botucatu, São Paulo, Brazil

**Keywords:** Vitamin D, chronic kidney disease, hemodialysis, mortality

## Abstract

**Introduction:**

Low vitamin D levels are associated with mortality in hemodialysis (HD) patients; however, the serum vitamin D thresholds are unclear. This study aimed to identify the vitamin D level below which mortality increases in HD patients.

**Methods:**

A cohort of HD patients enrolled from January 2014 to January 2017 was evaluated. The variables were analyzed according to the season, namely, summer, winter, and annual average, mortality was the primary outcome. The patients were assigned to vitamin D quintiles, and multivariate Cox regression analysis adjusted for age, ethnicity, gender, body mass index (BMI), inhibitors of the renin-angiotensin system, statin, calcitriol, and antiplatelet drugs use, hemodialysis vintage, hypertension, diabetes mellitus, atherosclerotic disease, and C-reactive protein was performed.

**Results:**

There were studied 306 patients. Vitamin D levels of 18.0–23.6 ng/mL (hazard ratio [HR] = 4.30; 95% confidence interval [CI] 1.60–11.54, *p* = 0.004) and <18.0 ng/mL (HR = 3.83; 95% CI: 1.42–10.35, *p* = 0.008) in summer and vitamin D levels of 21.5-27.1 ng/mL (HR = 3.70; 95% CI: 1.50-9.11, *p* = 0.004) and ≤17.5 ng/mL (HR = 2.84; 95% CI: 1.13–7.13, *p* = 0.026) in winter were associated with mortality. The average annual values of vitamin D associated with all-cause mortality were <17.7 ng/dL (adjusted HR = 4.25, 95% CI: 1.57–11.48, *p* = 0.004), and between >17.7 ng/dL and ≤23.1 ng/dL (adjusted HR = 3.91, 95% CI: 1.47–10.42, *p* = 0.006).

**Conclusions:**

Annual average vitamin D levels <23.1 ng/mL were associated with higher all-cause mortality, regardless of the confounding variables evaluated.

## Introduction

The number of patients on hemodialysis (HD) is increasing, and they have a high mortality rate [1], which is mainly a result of the presence of cardiovascular disease that is not completely explained by the presence of traditional risk factors [2]. HD patients often develop systemic mineral and bone metabolism disorders that involve biochemical and bone abnormalities, and vascular calcification [3–5]. These abnormalities are prevalent and represent nontraditional risk factors [6].

Most patients who undergo HD have vitamin D deficiencies, which can be influenced by age, skin pigmentation, the duration of exposure to the sun, obesity, the use of sunscreen, and the presence of diseases, including diabetes mellitus and chronic kidney disease (CKD) [7,8]. Up to 90% of HD patients can be vitamin D deficient, and this is associated with increased arterial stiffness, increases in the prevalence of vascular calcification, stroke, and left ventricular hypertrophy, and greater risks of all-cause death and cardiovascular mortality [2,9].

The findings from several observational studies have shown that low vitamin D levels are associated with all-cause mortality in HD patients [10,11]. A meta-analysis of prospective observational studies showed a reduced risk of mortality among patients with chronic renal disease and higher vitamin D levels, regardless of the disease stage [12].

Controversy exists regarding the optimal vitamin D level, and specific vitamin D levels for patient populations with and without CKD have not been defined [13]. The ideal serum 25-hydroxyvitamin D (25[OH]D) levels in patient populations should be stratified according to age and individual clinical characteristics. A serum 25(OH)D concentration >20 ng/mL is the reference level for healthy individuals ≤60 years old, and a concentration of 30–60 ng/mL is recommended for older adults, pregnant or lactating women, and for patients with rickets/osteomalacia, osteoporosis or secondary causes of osteoporosis, hyperparathyroidism, inflammatory diseases, autoimmune diseases, CKD, malabsorption syndromes, and patients with histories of falls and fractures [7].

The current CKD guidelines [14,15] recommend that, regardless of the CKD stage, 25(OH)D levels should be maintained at >30 ng/mL. However, the 25(OH)D level below which higher mortality rates occur in HD patients remains unclear. Thus, the objective of this study was to identify the serum 25(OH)D cutoff value below which mortality increases in HD patients.

## Methods

This unicentric cohort study enrolled patients who underwent HD from January 2014 to January 2017. The primary outcome was all-cause death, and the secondary outcome was death from cardiovascular causes. The end of follow-up was defined as death by any cause, withdraw of follow-up, or if do not reach any of these outcomes, followed until January/2017. This study was approved by the local research ethics committee (Approval number: 1622148), and the requirement for informed consent was waived because it was a retrospective study and it followed the Strengthening the Reporting of Observational Studies in Epidemiology Guidelines [16].

The variable of interest was the serum 25(OH)D level, which was measured by chemiluminescence immunoassay and evaluated every 6 months before the second session of hemodialysis, after 6 h of fasting, in the first weeks of January and July that represented summer and winter, respectively, in the southern hemisphere. The other variables evaluated included age, sex, race, body mass index (BMI), baseline diseases, arterial hypertension (characterized by sustained rise in blood pressure ≥ 140 and/or 90 mmHg), diabetes mellitus, atherosclerotic disease (encephalic vascular accident, coronary artery disease, and peripheral obstructive arterial disease), the time on HD, which was defined as the time from the onset of therapy to the first measurement of the serum 25(OH)D level, smoking, the arterial blood pressure, the laboratory test results, the use of calcitriol during the first year of follow-up, inhibitors of the renin-angiotensin system (RAS), statin, and antiplatelet drugs use, the fractional clearance of urea, and the vascular access type. The variables were analyzed according to the season, namely, summer, winter, and annual average, and the patients were compared in relation to all-cause death. The 25(OH)D levels were categorized into quintiles, because of the nonlinear associations with the outcomes.

HD patients were included if they were >18 years of age and had ≥1 laboratory test result for the serum 25(OH)D level. The exclusion criteria were a history of malignant neoplasms, parathyroidectomy, or hepatic cirrhosis, and patients who had undergone HD for <1 month.

### Statistical analyses

The data from the variables with normal distributions are expressed as the means and the standard deviations, and the data from the variables with nonnormal distributions are expressed as the medians and interquartile ranges. The frequencies are presented as percentages. Comparisons of the normally distributed data were performed using Student’s *t*-test, and comparisons of the nonnormally distributed data were performed using the Mann-Whitney test. The chi-squared test was used to analyze the categorical variables. Statistical inferences related to associations between the serum 25(OH)D levels and the outcomes were analyzed using multivariate Cox proportional hazards regression analyses. The variables that were associated with the outcomes at a level of significance of *p* < 0.1 in the univariate analysis were selected for the multivariate analysis. Gender, ethnicity, BMI, and medication use were forced into the model, because of the prognostic relevance of these variables. To assess the proportionality of the hazards in the Cox proportional hazards regression model, the Schoenfeld test was performed. A value of *p* < 0.05 was considered statistically significant. All of the statistical analyses were performed using IBM^®^SPSS^®^ software, version 20.0 for Windows (IBM Corporation, Armonk, NY, USA).

## Results

**Figure 1.**
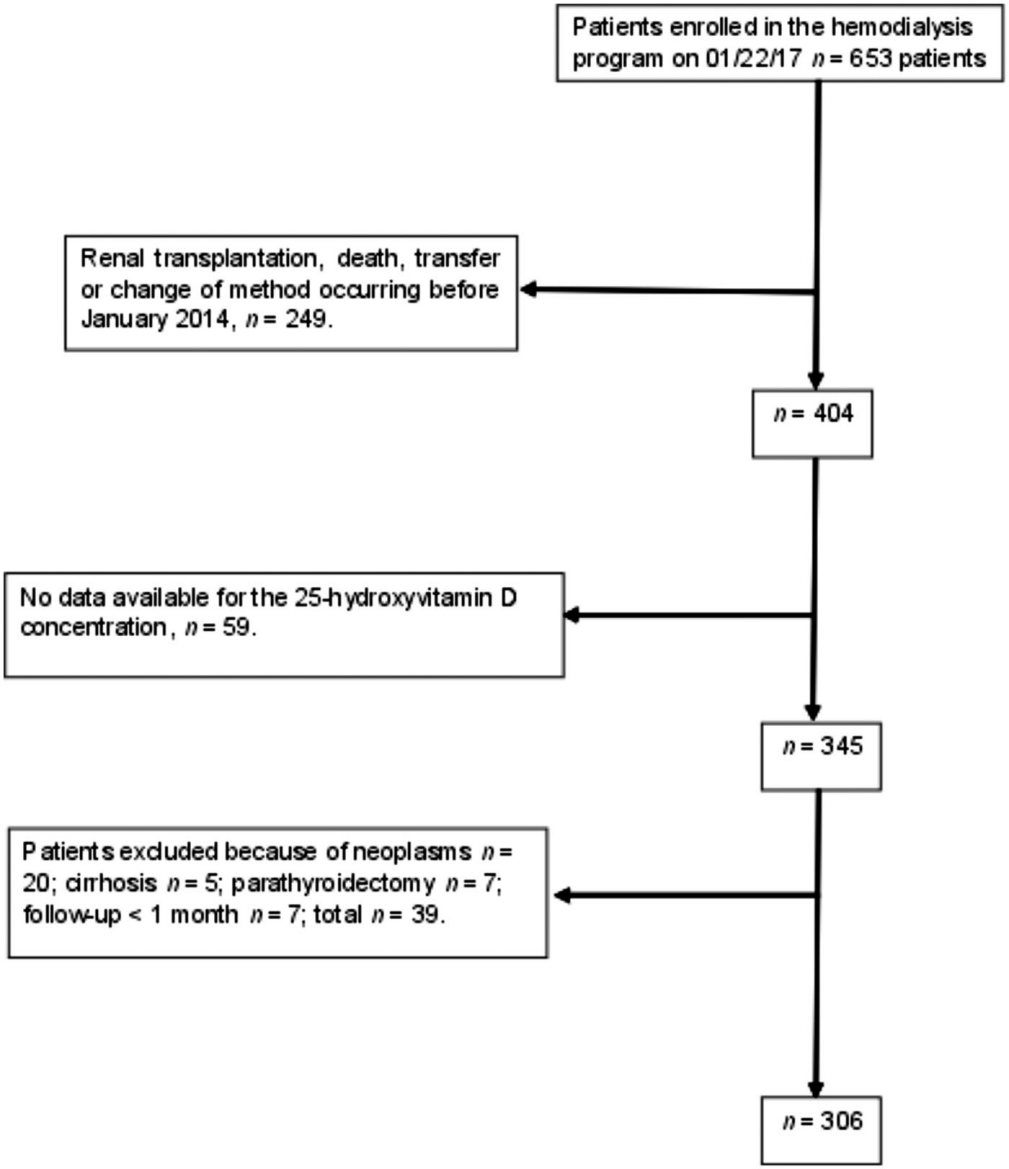
Study flowchart.

Figure 1 shows a flowchart of the patients’ inclusion in the study. No differences were evident between the patients who died or survived in relation to sex, race, BMI, and the type of vascular access. Statistically significant differences were evident between the patients who died or survived in relation to age, arterial hypertension, diabetes mellitus, atherosclerotic disease, HD vintage, and statin use (Table 1).

**Table 1.** Clinical characteristics of the patients on hemodialysis who survived or died.

	Total	Survivors	Nonsurvivors	*p* Value
	(*n* = 306)	(*n* = 233)	(*n* = 73)	
Age^+^ (years); mean (SD)	58 ± 15.0	56 ± 15.2	65 ± 12.3	<0.001
Sex* (%)				
Female	47	47	47	0.917
Male	53	53	53	
Ethnicity* (%)				
White	74	72	77	0.533
Mixed race	19	20	19	
Black	7	8	4	
CKD etiology* (%)				
Arterial hypertension	20	21	18	0.705
Diabetes	35	32	45	
Glomerular disease	11	13	4	
Urological disease	6	6.0	7	
Indeterminate	9	10	6	
Other causes^a^	19	18	20	
Comorbidity* (%)				
Arterial hypertension	82	78	93	0.006
Diabetes	47	42	60	0.012
Smoker	40	40	38	0.868
Atherosclerotic disease	39	33	38	<0.001
Other^b^	53	52	56	0.619
Median hemodialysis vintage** (months)	5.3 (2.3 − 26.8)	4.6 (2.0 − 21.1)	18.4 (3.8 − 33.4)	<0.001
Median follow-up duration (months)	18 (1 − 37)	18.6 (1 − 37)	13.6(1 − 36)	–
Weight (Kg)^+^	67.9 ± 15.91	68.4 ± 15.64	66.2 ± 16.72	0.298
Dry weight (Kg)^+^	67.0 ± 15.84	67.6 ± 15.48	65.0 ± 16.94	0.231
Height (cm)^+^	161 ± 9.8	161 ± 9.8	161 ± 9.87	0.713
BMI (kg/m^2^)^+^	25.9 ± 5.36	26.1 ± 5.26	25.3 ± 5.71	0.305
Vascular access (%)*				
Nontunneled catheter	8	6	14	0.900
Tunneled catheter	58	57	60	
Arteriovenous fistula	34	36	26	
Drugs use*				
Antiplatelet drugs	183	132	51	0.061
Statins	203	146	57	0.020
RAS inhibitors	129	96	33	0.639

SD: standard deviation; CKD: chronic kidney disease, RAS: renin-angiotensin system, BMI: body mass index. *Chi-squared test; ^+^*t*-test; **Mann–Whitney test.

^a^Other causes: ischemic nephropathy, polycystic kidney disease, acute renal injury, loss of renal graft, Alport’s syndrome, renal cystinosis; atherosclerotic disease: vascular accident encephalic, coronary artery disease, and peripheral obstructive arterial disease.

^b^Other comorbidities: dyslipidemia, hypothyroidism, obesity, cardiac arrhythmia, hyperuricemia, neurological, and others.

The number of vitamin D samples analyzed were from one per season (one in winter and one in summer) (only 76 patients) to four per season. The median of the number of repetitions was two per season. When there was more than one sample per season the vitamin D was averaged. The median time of follow-up was 18 months, from one to 36 months.

Regarding the laboratory test results (Table 2), the survivors and nonsurvivors differed in relation to the 25(OH)D and C-reactive protein levels in summer and winter, and they differed regarding the phosphorus and parathyroid hormone levels in winter. Of the 306 HD patients who participated in this study, 73 patients died. The main causes of death were infection (49.3%), cardiovascular (28.8%), undetermined (9.6%), and other (12.3%).

**Table 2.** Laboratory characteristics of the hemodialysis patients in summer and winter who survived or died.

Summer				
Variable	Total (*n* = 306)	Survivors (*n* = 233)	Nonsurvivors (*n* = 73)	*p* Value
Vitamin D (ng/mL); mean (SD)	27.6 ± 11.00	28.9 ± 10.88	23.3 ± 10.32	<0.001
Calcium (mg/dL); mean (SD)	8.7 ± 1.87	8.8 ± 1.76	8.6 ± 2.19	0.619
Phosphorus (mg/dL); mean (SD)	5.4 ± 1.72	5.4 ± 1.67	5.2 ± 1.88	0.363
PTH (pg/mL); median (range)	321 (188 − 569)	330 (197 − 582)	287 (135 − 526)	0.293
Albumin (g/dL); mean (SD)	3.9 ± 0.44	3.9 ± 0.45	3.9 ± 0.39	0.266
Phosphatase (µg/L); median (range)	111 (86 − 149)	110 (84 − 147)	112 (93 − 157)	0.272
CRP (mg/dL); median (range)	1.19 (0.70 − 2.10)	1.10 (0.67 − 2.00)	1.60 (0.90 − 3.07)	<0.001
Hemoglobin (g/dL); mean (SD)	11.5 ± 1.44	11.6 ± 1.48	11.4 ± 1.33	0.507
Ferritin (ng/mL); mean (SD)	801.5 ± 716.9	730.7 ± 671.9	872.4 ± 761.9	0.178
Iron (mcg/dL); mean (SD)	77.2 ± 36.7	78.2 ± 28.9	76.2 ± 44.6	0.700
Transferrin saturation (%); mean (SD)	35.4 ± 15.7	35.7 ± 13.0	35.1 ± 18.4	0.775
Pre-urea (mg/dL); mean (SD)	109.6 ± 30.5	110.3 ± 31.7	109 ± 29.3	0.784
Post-urea (mg/dL); mean (SD)	32.6 ± 12.8	33.1 ± 12.9	32.1 ± 12.8	0.603
URR; mean (SD)	0.70 ± 0.07	0.70 ± 0.07	0.70 ± 0.08	0.543
Kt/V; mean (SD)	1.26 ± 0.35	1.24 ± 0.30	1.29 ± 0.40	0.313

Winter				
Laboratory tests	Total (*n* = 306)	Survivors (*n* = 233)	Nonsurvivors (*n* = 73)	*p* Value
Vitamin D (ng/mL); mean (SD)	25.5 ± 8.95	26.3 ± 9.18	23.3 ± 7.89	0.024
Calcium (mg/dL); mean (SD)	9.1 ± 0.86	9.0 ± 0.90	9.2 ± 0.71	0.309
Phosphorus (mg/dL); mean (SD)	5.2 ± 1.81	5.4 ± 1.38	4.5 ± 2.51	<0.001
PTH hormone (pg/mL); median (range)	284 (154 − 495)	317 (179 − 566)	250 (102 − 504)	0.047
Albumin (g/dL); mean (SD)	3.9 ± 0.42	3.9 ± 0.41	3.8 ± 0.43	0.134
alkaline phosphatase (µg/L); median (range)	118 (90 − 153)	118 (88 − 154)	119 (94 − 153)	0.838
CRP (mg/dL); median (range)	1.60 (1.00 − 2.80)	1.50 (0.90 − 2.70)	2.15 (1.25 − 3.55)	0.009
Hemoglobin (g/dL); mean (SD)	11.3 ± 1.63	11.3 ± 1.40	11.3 ± 2.21	0.855
Ferritin (ng/mL); mean (SD)	793.8 ± 658.6	744.9 ± 636.5	842.8 ± 680.7	0.365
Iron (mcg/dL); mean (SD)	81.4 ± 30.7	81.5 ± 34.6	81.3 ± 26.9	0.962
Transferrin saturation (%); mean (SD)	35.5 ± 13.9	35.2 ± 13.8	35.8 ± 14.0	0.792
Pre-urea (mg/dL); mean (SD)	111.4 ± 28	114.6 ± 27.5	108.3 ± 28.6	0.169
Post-urea (mg/dL); mean (SD)	35.5 ± 14	35.6 ± 12	35.4 ± 16	0.914
URR; mean (SD)	0.67 ± 0.08	0.68 ± 0.06	0.67 ± 0.10	0.302
Kt/V; mean (SD)	1.17 ± 0.23	1.18 ± 0.19	1.16 ± 0.27	0.561

SD: standard deviation; PTH: parathyroid hormone; CRP: C-reactive protein; URR: urea reduction ratio; Kt/V: fractional clearance of urea.

For the survival analysis, the patients were assigned to quintiles according to their serum 25(OH)D levels in the summer (first quintile: ≤18.0 ng/mL; second quintile: >18.0–≤23.6 ng/mL; third quintile: >23.6–≤30.3 ng/mL; fourth quintile: >30.3–36.3 ng/mL; and fifth quintile: >36.3 ng/mL), winter (first quintile: ≤17.5 ng/mL; second quintile: >17.5–≤21.5 ng/mL; third quintile: >21.5–≤27.1 ng/mL; fourth quintile: >27.1–32.6 ng/mL; and fifth quintile: >32.6 ng/mL), and annual average (first quintile: ≤17.7 ng/mL; second quintile: >17.7–≤23.1 ng/mL; third quintile: >23.1–≤28.4 ng/mL; fourth quintile: >28.4– ≤34.8 ng/mL; and fifth quintile: >34.8 ng/mL). Twenty-one events relating to cardiovascular mortality occurred that were not associated with vitamin D in either summer or winter.

Table 3 presents the associations between the clinical variables evaluated in summer and mortality. We selected variables that were significant in the univariate analysis, and, after adjustments, the presence of diabetes mellitus, age, gender, ethnicity, calcitriol use during the first year of follow-up, antiplatelet drugs use, statin use, RAS inhibitors use, HD vintage, and C-reactive protein were excluded; and BMI, the presence of atherosclerotic disease and hypertension, and the 25(OH)D level remained in the model. The Schoenfeld proportionality was not statistically significant, which validated the use of the Cox regression model for the analysis.

**Table 3.** Cox regression analysis of the associations between the clinical variables in summer and mortality.

	HR	95% CI		*p* Value
		Lower	Upper	
Step 1				
Vitamin D quintile				
5th quintile: >36.3 ng/mL	1.000			
4th quintile: >30.3–≤36.3 ng/mL	2.378	0.829	6.821	0.107
3rd quintile: >23.6–≤30.3 ng/mL	1.755	0.540	5.704	0.350
2nd quintile: >18.0–≤23.6 ng/mL	4.136	1.391	12.292	0.011
1st quintile: ≤18.0 ng/mL	3.761	1.214	11.649	0.022
Age (years)	1.013	0,992	1.034	0.222
Gender	0.752	0.432	1.312	0.316
Ethnicity				
White	1.000			
Mixed race	1.332	0,672	2,642	0.411
Black	0,466	0,136	1,602	0.226
BMI (kg/m²)	0.947	0.899	0.998	0.042
Antiplatelet drugs use	0.792	0.440	1.425	0.437
Statin use	1.354	0.711	2.577	0.356
RAS inhibitors use	1.053	0.628	1.763	0.846
Calcitriol use in the first year of follow-up	0.928	0.542	1.588	0.784
Hemodialysis vintage (months)	1.002	0.996	1.008	0.533
Presence of hypertension	2.466	0.955	6.368	0.062
Presence of diabetes mellitus	1.566	0.874	2.807	0.132
Presence of atherosclerotic disease	1.423	0.834	2.429	0.195
CRP (mg/dL)	1.031	0.972	1.094	0.307
Step 6				
5th quintile: >36.3 ng/mL	1.000			
4th quintile: >30.3–≤36.3 ng/mL	2.474	0.885	6.917	0.084
3rd quintile: >23.6–≤30.3 ng/mL	1.677	0.544	5.167	0.368
2nd quintile: >18.0–≤23.6 ng/mL	4.302	1.603	11.541	0.004
1st quintile: ≤18.0 ng/mL	3.834	1.421	10.346	0.008
BMI (kg/m²)	0.952	0.909	0.997	0.035
Presence of hypertension	2.922	1.169	7.307	0.022
Presence of atherosclerotic disease	1.578	0.967	2.573	0.068

CRP: C-reactive protein; HR: hazard ratio; CI, confidence interval. BMI: body mass index; RAS: renin-angiotensin system.

During summer, significant associations were evident between all-cause mortality and the first 25(OH)D quintile (adjusted hazard ratio [HR] = 3.83, 95% confidence interval [95% CI]: 1.42–10.35, *p* = 0.008), all-cause mortality and the second 25(OH)D quintile (adjusted HR = 4.30, 95% CI: 1.60–11.54, *p* = 0.004), all-cause mortality and BMI (adjusted HR = 0.95, 95% CI: 0.90-0.99, *p* = 0.035), and between all-cause mortality and the presence of arterial hypertension (adjusted HR = 2.92, 95% CI: 1.17–7.30, *p* = 0.022). The presence of atherosclerotic disease was not associated with all-cause mortality (adjusted HR = 1.57, 95% CI: 0.96–2.57, *p* = 0.068).

Table 4 presents the associations between the clinical variables and all-cause mortality during winter. In this season, significant association were found between all-cause mortality and the first 25(OH)D quintile (adjusted HR = 2.84, 95% CI: 1.13–7.13, *p* = 0.026), all-cause mortality and the third 25(OH)D quintile (adjusted HR = 3.70, 95% CI: 1.50–9.11, *p* = 0.004). The presence of hypertension was independently associated with all-cause mortality (adjusted HR = 3.59, 95% CI: 1.29–9.97, *p* = 0.014).

**Table 4.** Cox regression analysis of the associations between the clinical variables in winter and mortality.

	HR	95% CI		*p* Value
		Lower	Upper	
Step 1				
Age (years)	1.020	0.996	1.045	0.100
Gender	0.965	0.530	1.758	0.907
Ethnicity				
White	1.000			
Mixedrace	1.765	0.856	3.640	0.124
Black	0.427	0.100	1.820	0.250
Presence of atherosclerotic disease	1.288	0.739	2.245	0.372
Hemodialysis vintage (months)	1.003	0.994	1.012	0.550
Presence of hypertension	3.102	1.066	9.027	0.038
Presence of diabetes mellitus	1.024	0.557	1.882	0.938
Phosphorus (mg/dL)	1.150	0.927	1.426	0.203
PTH hormone (pg/mL)	0.999	0.998	1.000	0.116
CRP (mg/dL)	1.067	0.997	1.142	0.062
Vitamin D quintiles				
5th >32.6 ng/mL	1.000			
4th >27.1-≤32.6ng/mL	1.850	0.675	5.072	0.232
3rd >21.5–≤27.1 ng/mL	3.260	1.265	8.401	0.014
2nd >17.5–≤21.5 ng/mL	1.688	0.599	4.760	0.322
1st ≤17.5 ng/mL	2.793	0.980	7.962	0.055
Step 7				
Presence of hypertension	3.591	1.292	9.978	0.014
PTH Vitamin D quintiles	0.999	0.998	1.000	0.096
5th >32.6 ng/mL	1.000			
4th >27.1-≤32.6ng/mL	1.900	0.704	5.126	0.205
3rd >21.5–≤27.1 ng/mL	3.699	1.503	9.106	0.004
2nd >17.5–≤21.5 ng/mL	1.831	0.710	4.725	0.211
1st ≤17.5 ng/mL	2.841	1.131	7.132	0.026

PTH: parathyroid hormone; CRP: C-reactive protein; HR: hazard ratio; CI, confidence interval.

As shown in Table 5, regarding the associations between clinical variables and mortality from all causes in the annual average, statistical significance were found between all-cause mortality and the presence of hypertension (adjusted HR = 2.75, 95% CI: 1.10–6.98, *p* = 0.031), between all-cause mortality and the first 25(OH)D quintile; <17.7 ng/dL (adjusted HR = 4.25, 95% CI: 1.57–11.48, *p* = 0.004), and between >17.7 ng/dL and ≤23.1 ng/dL (adjusted HR = 3.91, 95% CI: 1.47–10.42, *p* = 0.006).

**Table 5.** Cox regression analysis of the associations between the clinical variables in the annual average and mortality.

	HR	95% CI		*p* Value
		Lower	Upper	
Step 1				
Age (years)	1.022	1.000	1.043	0.049
Gender	1.255	0.722	2.181	0.421
Ethnicity				
White	1.000			
Mixedrace	2.485	0.573	10.778	0.224
Black	3.471	0.719	16.752	0.121
Presence of atherosclerotic disease	0.715	0.418	1.224	0.222
Hemodialysis vintage (months)	0.817	0.651	1.025	0.081
Presence of hypertension	2.099	0.812	5.428	0.126
Presence of diabetes mellitus	0.744	0.419	1.321	0.312
Phosphorus (mg/dL)	3.2 10^3^	0.564	1.83 10^3^	0.067
PTH hormone (pg/mL)	1.318	0.529	3.284	0.553
CRP (mg/dL)	0.273	0.066	1.140	0.075
BMI (kg/m²)	0.245	0.059	1.023	0.054
Antiplatelet drugs use	4.854	0.998	23.619	0.050
Statin use	2,474	0.509	12.031	0.262
RAS inhibitors use	3.373	0.752	15.123	0.112
Calcitriol use in the first year of follow-up	4.213	0.927	19.157	0.063
Vitamin D quintiles				
5th >34.8 ng/mL	1.000			
4th >28.4 ≤ 34.8 ng/mL	2.381	0.832	6.811	0.106
3rd >23.1 ≤ 28.4 ng/mL	1.865	0.603	5.767	0.279
2nd > 17.7 ≤ 23.1 ng/mL	3.540	1.243	10.082	0.018
1st ≤17.7 ng/mL	3.801	1.240	11.652	0.019
Step 7				
Age	1.018	0.999	1.037	0.065
Presence of hypertension	2.755	1.097	6.981	0.031
BMI (kg/m²)	0.956	0.913	1.001	0.056
Vitamin D quintiles				
5th >34.8 ng/mL	1.000			
4th >28.4 ≤ 34.8 ng/mL	2.648	0.942	7.445	0.065
3rd >23.1 ≤ 28.4 ng/mL	2.201	0.750	6.457	0.151
2nd >17.7 ≤ 23.1 ng/mL	3.917	1.473	10.419	0.006
1st ≤17.7 ng/mL	4.249	1.572	11.484	0.004

PTH: parathyroid hormone; CRP: C-reactive protein; HR: hazard ratio; CI, confidence interval; BMI: body mass index; RAS: renin-angiotensin system.

## Discussion

Several reasons underlie the presence of low serum 25(OH)D levels in CKD patients [17]. Hypovitaminosis D is harmful and providing vitamin D supplements can be beneficial [18]. There is disagreement in the literature regarding the benefits of vitamin D replacement therapy in patients with CKD who undergo HD, especially in relation to its effect on mortality [19]. In addition, the 25(OH)D target level that is associated with the best prognoses for these patients remains unclear [20]. This study’s findings showed an association between the serum 25(OH)D concentration in summer and mortality in HD patients, and we determined that a serum 25(OH)D level ≤23.6 ng/mL in summer was associated with a higher mortality rate, which indicates a possible minimum level that should be verified in subsequent studies. Notably, this association persisted even after adjusting the model for the confounding variables in the Cox proportional hazards regression analysis.

Association was also shown between serum 25 (OH) D concentration in winter and with higher mortality. However, this occurred in different quintile levels (first quintile and third quintile), with serum levels of 25 (OH) *D* ≤ 17.5 ng/mL and serum levels of 25 (OH) *D* > 21.5 – ≤27.1 ng/mL. However, no increased risk of death was found with the second quintile. We have no explanation for this finding, and there was a lack of information about this in the literature. We cannot conclude that these discrepancies occurred by chance. Therefore, the number of measures of vitamin D possibly could explain this paradoxical result, in the winter period. Due to the small sample size of observations for each patient group when they were divided into subgroups, we considered that we may have suffered a loss of statistical power. Probably, if we had a larger sample size, we could have had homogeneous results. Therefore, this study must be repeated with a larger sample size. If these data were reproduced in other cases, it would deserve pathophysiological studies.

Accounting for vitamin D levels that were annually averaged can produce more robust and precise results because the number of observations could be increased. In this study, regardless of the seasonal levels, the average annual value of vitamin D in HD patients that was associated with all-cause mortality was ≤23.1 ng/mL. Values that were very close to this were found in the analysis in the summer period. Thus, if we had to dose vitamin D only once a year, it would be preferred that this should be done in the summer period, because of the association with mortality.

Patients with CKD have elevated levels of factors associated with inflammation. Cholecalciferol supplementation reduces inflammation and, subsequently, the C-reactive protein level [21,22]. The findings from a randomized placebo-controlled clinical trial showed that cholecalciferol supplementation had an anti-inflammatory effect and it increased the expression of the intracellular vitamin D regulatory enzymes within lymphocytes in a uremic environment [23]. In the present study, the association between vitamin D and mortality persisted, even after the model was adjusted for the C-reactive protein levels. Therefore, the influence on mortality was inherent to these variables.

Unlike the findings from other observational studies [9,24], vitamin D was not associated with fatal cardiovascular events in this study; however, the number of these fatal events was small in this study, which may have contributed to the absence of an association. The findings from a systematic review of 13 randomized clinical trials that compared vitamin D supplementation with placebo, did not demonstrate that vitamin D supplementation modified the mortality or cardiovascular risk in patients with CKD [25].

The presence of atherosclerotic disease is a risk factor for mortality, and the association between the 25(OH)D concentration and vascular calcification could explain this phenomenon. The findings from a small observational study showed that the serum 25(OH)D concentration was negatively associated with the degree of vascular calcification in patients with end-stage CKD, indicating that vitamin D could, paradoxically, inhibit calcification [26].

In this study, the use of calcitriol during the first year of follow-up was not related to mortality; a result that differs from other studies’ findings reported in the literature. One study’s findings showed that treatment with calcitriol was associated with better survival among HD patients [27]. The findings from a systematic review of 14 observational studies demonstrated that using calcitriol was associated with reduced mortality [28]. However, these studies were prone to ‘immortal time bias’ [29], which describes a spurious association between exposure to a given drug and better survival that occurs as a consequence of survivors having longer follow-up durations in which the drug can be prescribed, and, in fact, it is not that survival is better among the patients who receive the drug, rather than the patients who survive the longest receive the drug at some point. In the current study, the use of calcitriol was taken into account only when it was used during the first year of follow-up, specifically to avoid immortal time bias. Hence, even though there were no associations between calcitriol use and the outcomes, this variable was included in the multivariate analysis and, even after this adjustment, the association between the 25(OH)D concentration and mortality persisted.

This study’s results should be considered in the context of its limitations. Given the inherent shortcomings associated with retrospective observational studies, this study’s design should be considered a limitation. Importantly, while the study sample was representative of Brazilian people undergoing HD in relation to age and sex, it was not representative of the underlying disease, because arterial hypertension is the main cause of CKD according to the 2016 Brazilian Society of Nephrology’s census [30], but in this study, the main cause of CKD was diabetes mellitus. Another factor associated with the representativeness of this study’s results was the high frequency of Caucasian participants. All of these differences reduce the external validity of this study’s data, and they probably limit the generalizability of the data to the Brazilian population south of Botucatu; therefore, the data generated by this study should be validated in prospective multicenter clinical studies that involve several regions in Brazil.

## Conclusion

An association between serum vitamin D levels and mortality in HD patients was identified. Vitamin D levels ≤23.6 ng/mL in the summer were associated with higher all-cause mortality rates as levels of 21.5–≤27.1 ng/mL and ≤17.5 ng/mL in the winter were associated with higher all-cause mortality. Regardless of seasonal levels, the average annual value of vitamin D in HD patients that was associated with all-cause mortality was ≤23.1 ng/mL.

This study will encourage further work to assess whether the reference vitamin D level currently used in the general population is applicable to HD patients. An interventional study involving different vitamin D targets is needed to assess its impact on mortality. The current evaluation has direct implications regarding the target vitamin D level that should be sought with cholecalciferol replacement therapy in these patients.
